# Enzymatic and Non-Enzymatic Antioxidant Responses of Young Tomato Plants (cv. Micro-Tom) to Single and Combined Mild Nitrogen and Water Deficit: Not the Sum of the Parts

**DOI:** 10.3390/antiox12020375

**Published:** 2023-02-04

**Authors:** Joana Machado, Marta W. Vasconcelos, Cristiano Soares, Fernanda Fidalgo, Ep Heuvelink, Susana M. P. Carvalho

**Affiliations:** 1 GreenUPorto—Sustainable Agrifood Production Research Centre/Inov4Agro, DGAOT, Faculty of Sciences, University of Porto, Campus de Vairão, Rua da Agrária 747, 4485-646 Vairão, Portugal; 2 CBQF—Centro de Biotecnologia e Química Fina—Laboratório Associado, Escola Superior de Biotecnologia, Universidade Católica Portuguesa, Rua Diogo Botelho 1327, 4169-005 Porto, Portugal; 3 Horticulture and Product Physiology Group, Department of Plant Sciences, Wageningen University, P.O. Box 16, 6700 AA Wageningen, The Netherlands; 4 GreenUPorto—Sustainable Agrifood Production Research Centre/Inov4Agro, Department of Biology, Faculty of Sciences, University of Porto, Rua do Campo Alegre s/n, 4169-007 Porto, Portugal

**Keywords:** combined abiotic stresses, enzymatic components, Micro-Tom cv, non-enzymatic components, oxidative stress biomarkers

## Abstract

This study aims to perform a broad analysis of the antioxidant (AOX) responses of young tomato plants exposed to single and combined mild nitrogen (N) and water deficits through the evaluation of oxidative biomarkers, non-enzymatic and enzymatic AOX components. ‘Micro-Tom’ seedlings were subjected to four treatments: control (CTR; 100%N + 100%W), N deficit (N; 50%N), water deficit (W; 50%W), and combined deficits (N + W; 50%N + 50%W). An enhancement of several non-enzymatic and enzymatic components was found in plants subjected to N + W deficit, which presented higher anthocyanins accumulation (up to 103%) as well as higher levels of superoxide dismutase (SOD) transcripts at root level and of ascorbate peroxidase (APX) and catalase (CAT) transcripts at shoot level. This increase in the gene expression was also translated in augmented SOD (up to 202%), APX (up to 155%) and CAT (up to 108%) activity compared to CTR plants and the single deficits. Overall, tomato plants were able to employ defense strategies to cope with this combined deficit, as demonstrated by the higher total AOX capacity (up to 87%) compared to the single deficits, which contributed to the maintenance of their redox homeostasis, with unchanged values of lipid peroxidation and hydrogen peroxide compared with CTR plants.

## 1. Introduction

The agricultural sector globally uses about 80% of the total available freshwater, as well as high amounts of nitrogen (N) fertilizers, with total required amounts projected to double by 2050 [1,2,3]. Therefore, new goals are being set to reduce the use of these inputs. This is the case of the recent European Green Deal (2020), which defined as targets a reduction of nutrient losses by at least 50% and the use of fertilizers by at least 20% by 2030 [4]. Despite the need to use fewer resources, it is important to take into account that water and N deficits significantly impact plants’ growth and metabolism, which will ultimately affect their productivity. Hence, it is important to understand the mechanisms involved in plant responses when subjected to these single or combined deficits in order to increase agricultural production in a sustainable way where the available resources are more efficiently used. Tomato has been often used as a model system to study these processes when plants are exposed to single abiotic stresses (recently reviewed by [5]).

One of the most crucial consequences of abiotic stress is the disturbance of the equilibrium between the generation of reactive oxygen species (ROS) and antioxidant (AOX) defense systems, which could result in a loss of cell viability and death [6,7]. Drought impairs plant growth, affecting several physiological and biochemical processes, such as photosynthesis, respiration, nutrient uptake, and photoassimilates translocation [8]. N supply is crucial for plant development and growth, because it affects almost all cellular processes, from the production of metabolites, carbohydrates, fats, and pigments to general protein synthesis [9,10]. Therefore, water, as well as N shortage, can ultimately lead to reduced yield, loss of viability, and redox status alterations [11]. The cellular damage caused by water deficit has two primary causes: first, the overproduction of ROS, and second, the alteration of water relationships within the plant [6]. Free ROS may then attack biological structures, damaging DNA, prompting the oxidation of amino acids and proteins, and provoking lipid peroxidation [7]. To avoid such damage, plants have developed ROS-detoxification mechanisms that work in tandem to counteract ROS overproduction [6,7]. Several studies using different tomato cultivars showed that drought stress increases the production of ROS and the oxidative load, including AOX substrates and AOX enzymes [12,13]. Little is known about the effects of N deficiency on ROS content and AOX enzyme activity in tomato plants, but some studies suggest that ROS content increases in tomato as a result of N starvation, as demonstrated by higher levels of malondialdehyde (MDA) in the root system of N-deficient tomato [14].

Moreover, understanding how plants regulate their AOX defenses under a combined N and water deficit situation is important, as crops are often exposed to a combination of these two stresses under field conditions [15]. When two stresses occur concurrently, the plant’s acclimation strategy is governed by the interaction of the two stresses, which is conceived by the plant as a new state of stress [16]. Rivero et al. [17] and Sousa et al. [18] demonstrated that tomato plants grown under the combination of salinity and heat stress showed a profile of accumulation of osmoprotectants and AOX metabolites that was not observed when these stresses were applied individually. Martinez et al. [19] also demonstrated that the combination of salinity and heat increases tomato metabolomic profile differently from when these stresses were applied individually. This demonstrates that the combination of two or more abiotic stressors induces a unique plethora of responses that cannot be extrapolated from those observed under single exposure [20,21]. Based on these premises, this study aimed to unravel the AOX responses of tomato seedlings to single and combined N and W deficits and to determine the differences when these stressors occur individually. To that end, oxidative stress biomarkers (lipid peroxidation (MDA equivalents) and hydrogen peroxide (H_2_O_2_) levels) were evaluated, along with the assessment of non-enzymatic (ABTS (2,2′-azino-bis(3-ethylbenzothiazoline-6-sulfonic acid), phenolics, flavonoids, and anthocyanins) and enzymatic (superoxide dismutase—SOD; catalase—CAT; ascorbate peroxidase—APX) AOX components, both at transcript and enzyme activity levels.

## 2. Materials and Methods

### 2.1. Plant Material and Growth Conditions

The experiment was carried out in a growth chamber (temperature: 25 °C day/23 °C night; relative humidity: 70%; photosynthetic photon flux density (PPFD): 300 µmol s^−1^ m^−2^; photoperiod: 16 h light).

*Solanum lycopersicum* cv. ‘Micro-Tom’ seeds were sown in trays filled with a commercial germination potting substrate (SIRO, Portugal). At third leaf appearance (approximately three weeks after sowing), seedlings were selected for uniformity and transferred to single-plant pots (10 cm high, 10 cm diameter) filled with 0.1 to 1.5 mm grade vermiculite (60 g) and divided into four experimental groups: control (CTR; 10.5 mM N 100% field capacity), nitrogen deficit (N; 5.3 mM N), water deficit (W; 50% field capacity) and combined deficit (N + W; 5.3 mM N; 50% field capacity). Since we aimed at having a progressive water deficit, as seedling transplanting is a critical phase of the cultivation period, just prior to transplanting, all pots were irrigated to field capacity (FC), determined using the soil gravimetric water content method [22], by adding ~270 mL of the respective nutrient solution (CTR and W deficit treatments: 10 mM NO_3_^−^; 0.5 mM NH_4_^+^; 1.9 mM H_2_PO_4_; 6.1 mM K^+^; 3.6 mM Ca^2+^; 1.6 mM SO_4_^2^; 2.5 mM Mg^2+^; 2.6 mM Cl^−^; 0.5 mM HCO_3_^−^ (pH 6 and electrical conductivity—EC—of 2.50 dSm^−1^); N deficit and N + W deficit treatments: 5 mM NO_3_^−^; 0.3 mM NH_4_^+^; 1.9 mM H_2_PO_4_; 6.1 mM K^+^; 3.6 mM Ca^2+^; 4.8 mM SO_4_^2^; 2.8 mM Mg^2+^; 5.5 mM Cl^−^; 0.5 mM HCO_3_^−^ (pH 6 and EC of 2.80 dSm^−1^), with both solutions having the same micronutrients composition (35 μM Fe-EDDHA; 10 μM Mn^2+^; 20.1 μM B; 0.9 μM Cu^2+^; 5.0 μM Zn^2+^; 0.5 μM MoO_4_^2^)).

Following seedling transplantation, all pots were covered to prevent evaporation and no more nutrient solution was added until the end of the experiment. Concerning further irrigation, each pot from CTR and N deficit treatment was weighted and re-watered (with distilled H_2_O) on a daily basis, to maintain FC at 100% during the experimental period, whereas in W and N + W deficit treatments, no additional irrigation was supplied, resulting in a progressive decrease in substrate FC. The experiment ended when plants from treatments W and N+W reached 50% of FC (day 16). At this point, six plants from each treatment were harvested and used for the analysis of morphological and physiological parameters. The remaining plants were separated into roots and shoots, and immediately frozen in liquid nitrogen, macerated (*n* = 3, each biological replicate consisting of a pool of three individual plants) and, subsequently, stored at −80 °C for further use in biochemical and molecular analyses.

### 2.2. Biometric Traits

At the end of the experimental period (i.e., 16 days after transplantation), 6 plants per treatment were divided into roots, stems, and leaves. After carefully washing the roots, the dry weight of each organ was determined, by drying the samples for 48 h at 105 °C in a ventilated oven. Based on these data, the shoot–root ratio was further calculated (*n* = 6).

### 2.3. Analysis of Total Antioxidant Capacity and Nonenzymatic Component

#### 2.3.1. Preparation of the Extracts

For the analysis of the total antioxidant activity (ABTS^•+^ assay) and secondary metabolism (total soluble polyphenols, flavonoids, anthocyanins, and lignin), 300 or 250 mg of frozen grinded samples of roots and shoots, respectively, were extracted with 1.2 or 1.5 mL of 80% (*v*/*v*) methanol, respectively, under cold conditions. Samples were centrifuged at 15,000× *g* for 15 min at 4 °C. The resulting supernatant was used for all previously mentioned analyses, except lignin quantification, where the sediment was used.

#### 2.3.2. Total Antioxidant Capacity: ABTS Assay

ABTS^•+^ (2,2-azinobis-(3-ethylbenzothiazoline-6-sulphonic acid)) assay (*n* = 3) was performed following the protocol described by Gonçalves et al. [23], with slight modifications for the 96-well microplate. Briefly, ABTS^•+^ solution was generated through a chemical oxidation reaction with potassium persulfate and its concentration was adjusted with methanol to an initial absorbance of 0.700 (±0.020) at 734 nm (Multiskan GO Microplate Spectrophotometer, Thermo FisherScientific Inc., Waltham, MA, USA). To 20 µL of sample or standard solution, 180 μL of the adjusted ABTS^•+^ solution was added. The mixture was then incubated for 5 min at 30 °C, the absorbance at 734 nm was measured and the results were obtained by calculating the reduction of percentage in sample absorbance (A_SPL_) with respect to the control (A_CTL_) using Equation (1). Trolox was used as a standard to prepare a calibration curve, and the final results are expressed as mmol Trolox equivalents/g FW. All the analyses were performed in triplicate, and the radical stock solution was freshly prepared and filtered with a 0.45 µm syringe filter.

(1) $$ABTS\;scavenging\left(\%\right)=\left(A_{CTL}-A_{SPL}\right)*\frac{100}{A_{CTL}}$$

#### 2.3.3. Non-Enzymatic Component: Total Phenolics, Flavonoids, Carotenoids, Anthocyanins, and Lignin

##### Determination of Total Phenolic Content (TPC)

Determination of TPC was performed using the Folin–Ciocalteu reagent following Ramos et al. [24], with slight modifications. Briefly, 20 µL of extract or standard (gallic acid stock solutions) was mixed with 80 µL of Folin–Ciocalteu reagent (previously diluted 1:10 (*v*/*v*)) and 100 μL of 7.5% (*w*/*v*) sodium carbonate in a 96-well plate. After 60 min at room temperature under dark conditions, absorbance was measured at 750 nm. The results are expressed as mg gallic acid equivalents/g of fresh weight (mg GAE/g FW).

##### Determination of Total Flavonoids

Total flavonoids were quantified through the aluminum chloride method adapted from Zafar et al. [25]. Briefly, an aliquot of each extract (40 μL) was mixed with 20 μL of 10% (*w*/*v*) aluminum chloride and 20 μL of 1 M potassium acetate solutions. Thereafter, distilled water was added to obtain a final volume of 200 μL in a 96-well plate. After 30 min of incubation under dark conditions at room temperature, the absorbance at 415 nm was measured by using a microplate reader. The calibration curve was drawn by using quercetin as standard and the flavonoid concentration was established in μg quercetin equivalents/g FW (μg QE/g FW).

##### Determination of Total Monomeric Anthocyanins

The spectrophotometric pH differential method was used to determine the total monomeric anthocyanins (TMA) in the methanol extracts, following Nicoué et al. [26] with some modifications for the 96-well microplate. Briefly, 240 μL of 0.025 M potassium chloride solution or 0.4 M sodium acetate solution adjusted to pH 1.0 and 4.5, respectively, with hydrochloric acid were added to 60 μL of extracted sample. The absorbance was measured at 520 nm and 700 nm and the absorbance (A) was calculated as described in Equation (2):

(2) $$A={\left(A_{\lambda max}-A_{700}\right)}_{pH=1.0}-{\left(A_{\lambda max}-A_{700}\right)}_{pH=4.5}$$

TMA concentration was then obtained using Equation (3):

(3) $$TMA\left(mg/L\right)=\left(A_{CTL}-A_{SPL}\right)*\frac{A*MW*DF*1000}{\varepsilon *l}$$

where

λ_max_ = 520 nm

MW of cyanidin 3-glucoside = 449.2 g mol^−1^

DF = dilution factor

*ε* of cyanidin 3-glucoside = 26,900 L mol^−1^ cm^−1^

l = optical path length

##### Determination of Lignin

Lignin quantification was performed according to a protocol adapted to 96-well microplate from Fukushima and Hatfield [27]. Briefly, the sedimented biomass that remained in the microcentrifuge tube after methanol extraction was subjected to three 20 min washes in an ultrasound bath first with distilled water, then with acetone, and finally with hexane. Afterward, samples were dried at 60 °C for 72 h, and to 10 mg of sample, 1 mL of 12.5% acetyl bromide (prepared in acetic acid) was added, followed by incubation at 50 °C for 2 h with vigorous stirring. The digested samples were centrifuged for 5 min at 15,000× *g*, and 100 μL of the supernatant were combined with 200 μL of acetic acid, 150 μL of 0.3 M sodium hydroxide, 50 μL of 0.5 M hydroxylamine hydrochloride and 500 μL of acetic acid. The absorbance of each sample was recorded at 280 nm, and the lignin concentration in each sample was quantified taking into consideration the lignin calibration curve, and the lignin concentration was established in μg quercetin equivalents/g FW (μg/g FW).

### 2.4. Oxidative Stress Biomarkers

#### 2.4.1. Lipid Peroxidation

Lipid peroxidation (LP) was estimated in frozen ground samples of roots and shoots (ca. 200 mg FW) and quantified following the procedure of Heath and Packer [28]. After homogenizing the material in 0.1% (*w*/*v*) trichloroacetic acid, malondialdehyde (MDA) content was determined using the molar extinction coefficient (ε) of 155 mM^−1^ cm^−1^ and expressed in nmol^−1^ g FW. Absorbances were read at 532 and 600 nm, with the latter being subtracted from the first to avoid the effects of non-specific turbidity.

#### 2.4.2. Hydrogen Peroxide (H_2_O_2_) Quantification

The quantification of H_2_O_2_ levels was performed by a spectrophotometric method according to Alexieva et al. [29]. Briefly, frozen ground samples of roots and shoots (ca. 200 mg FW) were extracted on ice, with 1.4 mL of 0.1% (*w*/*v*) trichloroacetic acid, and centrifuged for 15 min at 12,000× g at 4 °C. The homogenate was then mixed with 250 μL of 100 mM potassium phosphate buffer (pH 7.0) and 1 mL of 1 M potassium iodide. The reaction was developed for 1 h in dark and absorbance was measured at 390 nm. H_2_O_2_ levels were estimated from a standard curve prepared with solutions of known concentration of H_2_O_2_. The results are expressed as nmol H_2_O_2_ g^−1^ FW.

### 2.5. Enzymatic Component

#### 2.5.1. Enzyme Extraction and Quantification: SOD (EC.1.15.1.1), CAT (EC.1.11.1.6) and APX (EC.1.11.1.11)

The extraction of the AOX enzymes was performed under cold conditions according to Fidalgo et al. [30], using frozen samples of around 250 mg. After centrifugation, the supernatants were collected and used for both protein and enzyme quantification following the protocols described by Soares et al. [31]. Briefly, protein quantification was performed as described by Bradford [32], using bovine serum albumin as standard. Superoxide dismutase (SOD, EC.1.15.1.1) activity was quantified with a spectrophotometric assay based on the inhibition of photochemical reduction of nitroblue tetrazolium (NBT) [33]. SOD activity was expressed as units of SOD mg^−1^ protein, in which one unit of SOD corresponds to the amount of enzyme needed to inhibit the photochemical reduction of NBT by 50% [21]. Absorbance was read at 560 nm and results are expressed as units of SOD/mg of protein. Catalase (CAT, EC.1.11.1.6) activity was determined spectrophotometrically by measuring CAT-mediated H_2_O_2_ degradation (ε_240nm_ = 43.6 mM^−1^ cm^−1^) [34]. Absorbance measurements were recorded every 5 s for 60 s, and results are expressed as mmol H_2_O_2_ min^−1^ mg of protein. Ascorbate peroxidase (APX, EC.1.11.1.11) activity was determined by monitoring the oxidation of ascorbic acid (AsA) for 1 min, with readings every 5 s (ε_290nm_ = 0.49 M^−1^ cm^−1^) [35]; results are expressed as mmol dehydroascorbate (DHA) min/mg of protein. The protocols for quantifying CAT and APX activity were adapted to UV microplates following the adaptation from Sousa et al. [31].

#### 2.5.2. SOD, APX, and CAT Expression Profile

The transcript levels of *Fe-SOD*, *Cu,Zn-SOD*, *APX*, *CAT1*, and *CAT2* were evaluated using quantitative real-time PCR.

Total RNA was extracted from frozen grinded roots and shoot tissues (c.a. 100 and 50 mg) using the RNeasy Plant Mini Kit (Qiagen, Hilden, Germany), according to the manufacturer’s instructions for each tissue type. Afterward, RNA yield was quantified using a NanoPhotometer (Implen GmbH, Germany), and its integrity was checked by agarose gel electrophoresis. Single-stranded cDNA was synthesized using iScript cDNA Synthesis Kit (Bio-Rad, Hercules, CA, USA) according to the manufacturer’s instructions in a Doppio Thermal Cycler (VWR, Oud-Heverlle, Belgium).

Primers for *APX*, *CAT1*, and *CAT2* were selected using Primer-BLAST from NCBI (https://www.ncbi.nlm.nih.gov/tools/primer-blast/, accessed on 15 May 2021) for an expected PCR product of 70–150 bp and primer melting temperatures between 57 and 63 °C, whereas primer sequences for *Fe-SOD* and *Cu-Zn-SOD* were obtained from previous studies [36]. The primer sequences for each gene are listed in Table 1.

Real-time polymerase chain reactions (RT-qPCR) were performed on a StepOne™ Real-Time PCR System (Applied Biosystems, California, USA) with the following reaction conditions: 2 min at 50 °C, 2 min at 95 °C and 40 cycles, with 15 s at 95 °C, 15 s at each primer pair optimal annealing temperature (Table 1) and 1 s at 72 °C. Amplifications were carried out using a final volume of 20 μL, which consisted of 1 μL of the primer forward and 1 μL of the primer reverse (both at 6 μM), 10 μL of 2× iQ SYBR^®^ Green Supermix (Bio-Rad, California, USA), and 8 μL of a 1:100 dilution of the template cDNA. Melt curve profiles were analyzed for each tested gene. The comparative CT method (^ΔΔ^CT [37]) was used for the relative quantification of gene expression values using ubiquitin (UBI) and TIP41-like protein (TIP41) genes as control transcript and the control plants as the reference sample. For relative gene expression analysis, the fold change with biological significance was considered to be lower than 0.5 (downregulation) or higher than 2 (upregulation) [38], and for each of the three biological replicates and target genes, two technical replicates were analyzed.

### 2.6. Statistical Analysis

Data were subjected to analyses of variance (ANOVA), followed by Tukey’s post hoc test, whenever significant differences were found (*p* < 0.05). The results are expressed as mean ± standard error of the mean (SEM). Additionally, a Principal Component Analysis (PCA) with Varimax rotation was used to establish the relationship among the different quantitative variables with the data set including only the parameters that were shown to be significantly different among treatments. The data included was mean-centered and weighed with the inverse of the standard deviation before applying the PCA to guarantee that all variables had the same weight in influencing the model. All statistical analyses were performed in IBM SPSS Statistics 26.

## 3. The Results

### 3.1. Plant Growth

**Figure 1:**
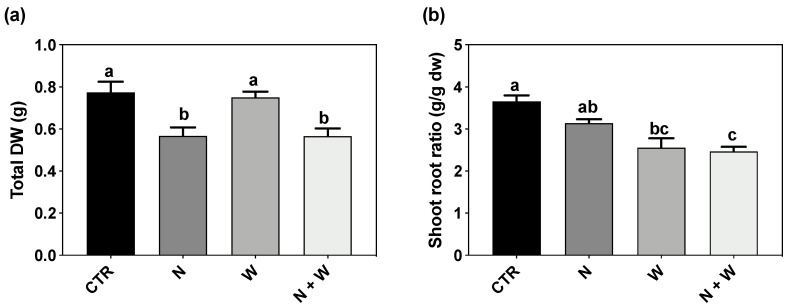
Total dry weight (**a**) and shoot–root ratio (**b**) of tomato plants cv. Mico-Tom grown for 16 days under control (CTR; 10.5 mM N + 100% W), nitrogen deficit (N; 5.3 mM N + 100%W), water deficit (W; 10.5 mM N + 50% W) or combined nitrogen and water deficit (N + W; 5.3 mM N + 50% W). Data presented are mean ± SEM (*n* = 6). Different letters above bars indicate significant differences according to the Tukey’s HSD test (*p* = 0.05).

The total dry weight (TDW) of plants grown under single or combined N deficit was significantly lower (30%) when compared to CTR and W-deficit plants. (Figure 1a). The shoot–root ratio of N + W deficit plants was 22% lower compared to plants grown under single N deficit. However, it did not significantly differ from plants grown under single W deficit. For the single deficits, this ratio was only significantly changed under W shortage, which led to a 30% decrease compared to CTR plants (Figure 1b).

### 3.2. AOX Activity

**Figure 2:**
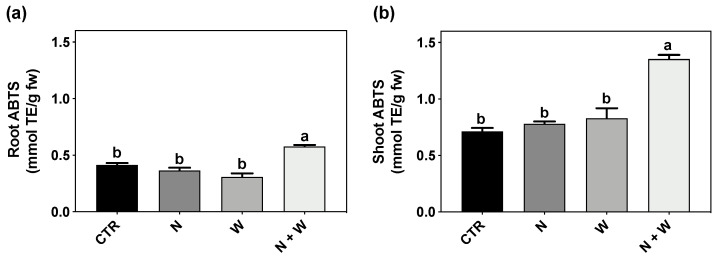
Total antioxidant capacity measured by ABTS in roots (**a**) and shoots (**b**) of tomato plants cv. Micro-Tom grown for 16 days under control (CTR; 10.5 mM N + 100% W), nitrogen deficit (N; 5.3 mM N + 100%W), water deficit (W; 10.5 mM N + 50% W) or combined nitrogen and water deficit (N + W; 5.3 mM N + 50% W). Data presented are mean ± SEM (*n* = 3). Different letters above bars indicate significant differences according to Tukey’s HSD test (*p* = 0.05).

Total AOX activity, measured using the ABTS assay, was significantly increased by about 90% in plants exposed to combined N + W deficit, in both roots and shoots, compared to all other experimental conditions, which displayed no differences among them (Figure 2a,b).

### 3.3. Non-Enzymatic Component: Total Phenolics, Flavonoids, Carotenoids, Anthocyanins, and Lignin

**Figure 3:**
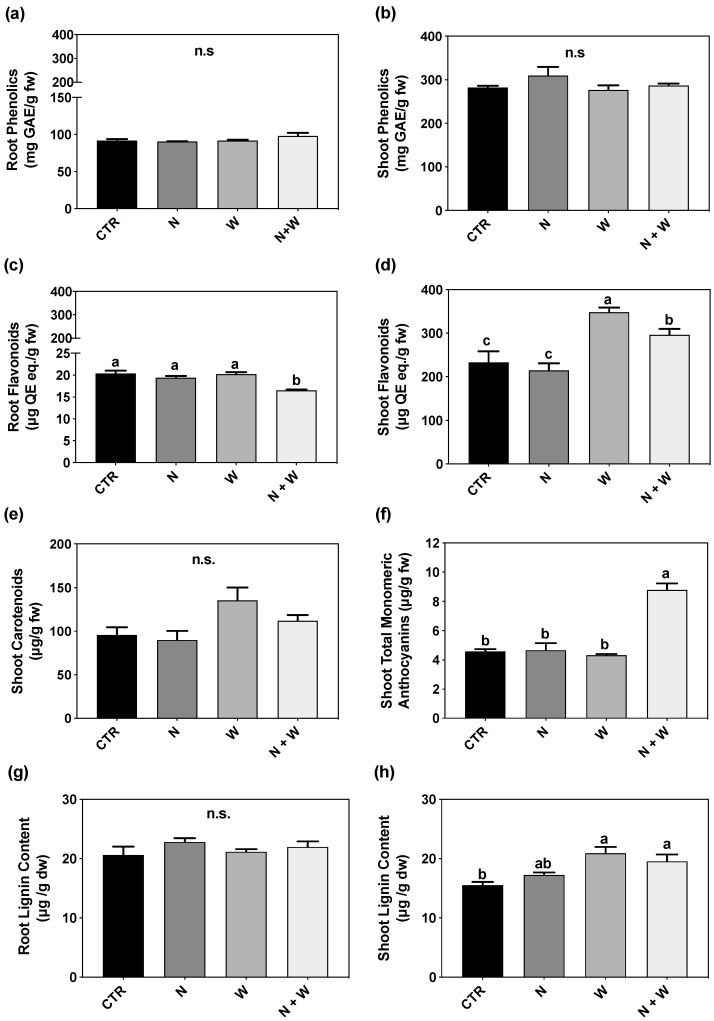
Total phenolics in roots (**a**) and shoots (**b**), total flavonoids in roots (**c**) and shoots (**d**), total monomeric anthocyanin in roots (**e**) and shoots (**f**), and lignin concentration in roots (**g**) and shoots (**h**) of tomato plants cv. Micro-Tom grown for 16 days under control (CTR; 10.5 mM N + 100% W), nitrogen deficit (N; 5.3 mM N + 100%W), water deficit (W; 10.5 mM N + 50% W) or combined nitrogen and water deficit (N + W; 5.3 mM N + 50% W). Data presented are mean ± SEM (*n* = 3). Different letters above bars indicate significant differences and n.s. correspond to non-significant differences between treatments according to Tukey’s HSD test (*p* = 0.05).

The concentration of total phenolics in roots and shoots was not affected by single or combined N + W deficit (Figure 3a,b). However, flavonoid concentration was reduced by around 20% in the roots of plants exposed to combined N + W deficit in relation to CTR or to single deficits (Figure 3c). Interestingly, shoot flavonoid concentration was significantly higher in single W deficit plants, as compared to the remaining experimental conditions, whereas plants subjected to the combined deficit had intermediate levels (17% lower compared to plants grown under W deficit and 15% higher compared to single N deficit) (Figure 3d). The levels of shoot carotenoids were not significantly affected. Still, a clear tendency could be observed, with W deficit plants having the highest levels of carotenoids (Figure 3e). The concentration of total monomeric anthocyanins reached the highest level in plants grown under combined N + W deficit, compared to all the other experimental groups (92%—CTR; 89%—N; 103%—W increase) (Figure 3f). Lignin concentration in the roots was not affected by any treatment (Figure 3g). In the shoots, it was significantly higher in plants exposed to W (by 35%) and N + W (by 26%) deficits compared to the CTR plants, but no significant differences between single and combined deficits were found (Figure 3h).

### 3.4. Oxidative Stress Biomarkers—MDA and H_2_O_2_ Levels

**Figure 4:**
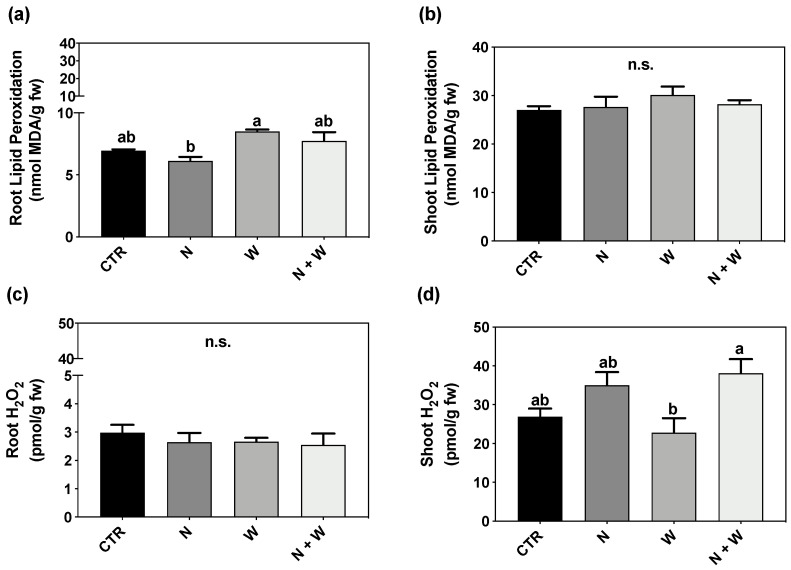
Lipid peroxidation of roots (**a**) and shoots (**b**) and H_2_O_2_ content of roots (**c**) and shoots (**d**) of tomato plants cv. Micro-Tom grown for 16 days under control (CTR; 10.5 mM N + 100% W), nitrogen deficit (N; 5.3 mM N + 100% W), water deficit (W; 10.5 mM N + 50% W) or combined nitrogen and water deficit (N + W; 5.3 mM N + 50% W). Data presented are mean ± SEM (*n* = 3). Different letters above bars indicate significant differences, and n.s. corresponds to non-significant differences between treatments according to Tukey’s HSD test (*p* = 0.05).

The MDA concentration in roots and shoots was not significantly influenced by the imposed deficits (Figure 4a,b). H_2_O_2_ concentration was not affected in roots (Figure 4c); however, in shoots, H_2_O_2_ concentration was 40% higher in plants exposed to N + W deficit than in plants subjected to W deficit (Figure 4d).

### 3.5. Enzymatic Component: SOD, CAT, APX

**Figure 5:**
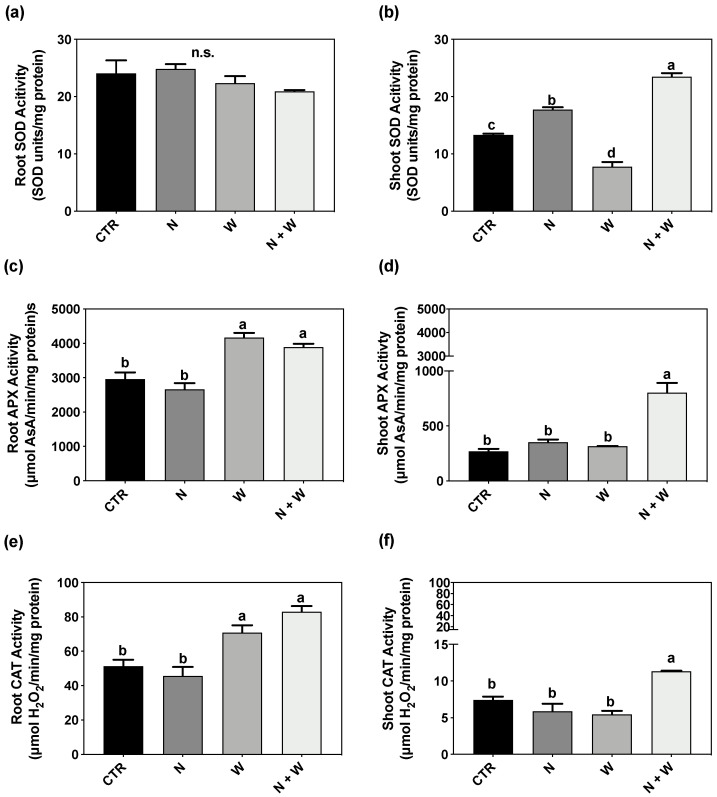
SOD activity in roots (**a**) and shoots (**b**), APX activity in roots (**c**) and shoots (**d**) and CAT activity in roots (**e**) and shoots (**f**) of tomato plants grown for 16 days under control (CTR; 10.5 mM N + 100% W), nitrogen deficit (N; 5.3 mM N + 100%W), water deficit (W; 10.5 mM N + 50% W) or combined nitrogen and water deficit (N + W; 5.3 mM N + 50% W). Data presented are mean ± SEM (*n* = 3). Different letters above bars indicate significant differences and n.s. correspond to non-significant differences between treatments according to the Tukey’s HSD test (*p* = 0.05).

In roots, SOD activity was not significantly affected by the imposed stresses (Figure 5a). In shoots, significant differences were found among all the treatments. N + W plants had the highest SOD activity (77%—CTR; 32%—N; 202%—W increase), and W deficit plants had the lowest SOD activity (Figure 5b). APX activity was significantly higher (up to 57%) in the roots of W and N + W deficit plants compared to CTR and single N deficit (Figure 5c). In shoots, APX activity was higher in N + W plants compared to CTR (by 198%), N (by 128%), and W (by 155%) (Figure 5d). CAT activity followed the same trend as APX, both in roots and shoots (Figure 5e,f).

### 3.6. SOD, APX, and CAT Expression Profile

**Figure 6:**
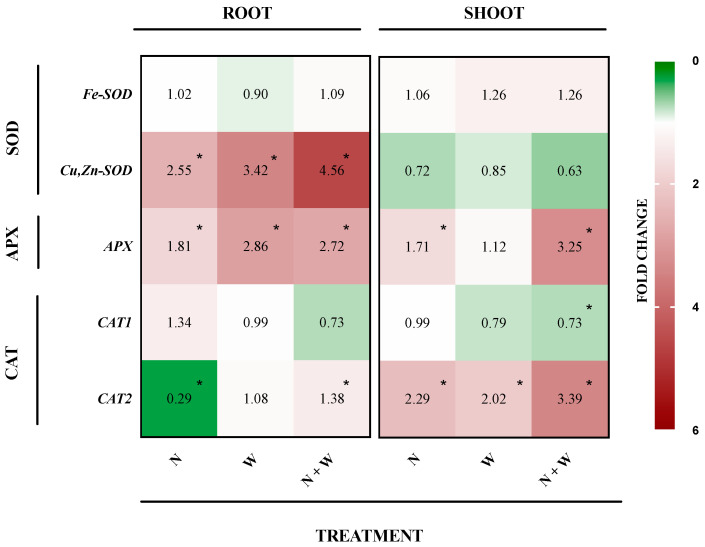
Relative fold of expression of antioxidant system-related genes in tomato (cv. Micro-Tom) roots and shoots as affected by nitrogen deficit (N; 5.3 mM N + 100% W), water deficit (W; 10.5 mM N + 50% W) or combined nitrogen and water deficit (N + W; 5.3 mM N + 50% W). The 2−∆∆Ct values against the control are shown (*n* = 3). * Values indicate significant differences compared to control according to the t-test (*p* = 0.05). Green color indicates downregulation and red color upregulation compared to the control plants.

*Fe-SOD* expression levels, in both roots and shoots of all treatments, were maintained within the basal threshold (with a fold of expression between 0.9 and 1.3) (Figure 6). However, in roots, a significant upregulation of *Cu,Zn-SOD* was observed for all deficits, with the levels being higher in the combined N + W deficit group (4.6-fold upregulation). In shoots, no significant differences were found in the expression of this gene compared to CTR plants (Figure 6). Single as well as combined deficit resulted in a significant increase in root *APX* expression, with the highest being in W (by 2.9-fold) and N + W (by 2.7-fold) plants. In shoots, N + W showed the highest expression of *APX* (by 3.3-fold), followed by N deficit plants (upregulation of 1.7-fold) (Figure 6). *CAT1* expression was maintained at basal levels in all deficit treatments both in the roots and the shoot, whereas *CAT2* expression was significantly downregulated in roots to N deficit plants (by 0.3-fold), but upregulated in shoots of all the treatments when compared to CTR. This was more pronounced in the N + W plants, which showed a 3.4-fold upregulation (Figure 6).

### 3.7. Principal Component Analysis (PCA)

**Figure 7:**
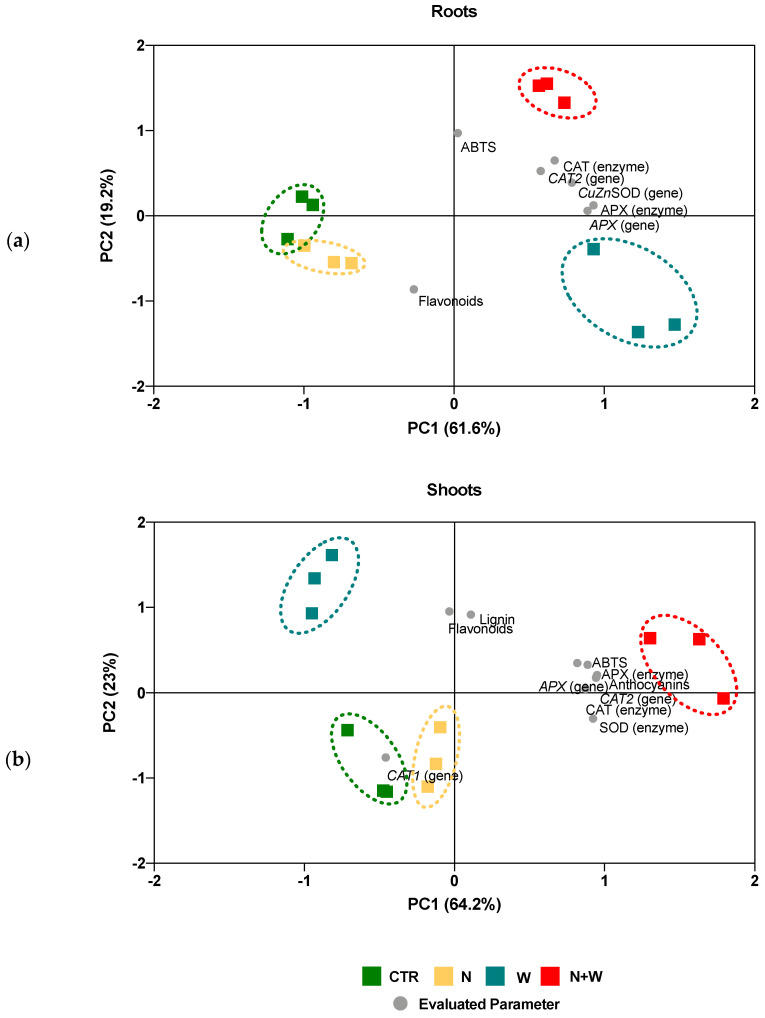
Principal component analysis (PCA) of the antioxidant system in roots **a**) and shoots (**b**) evaluated in tomato plants cv. Micro-Tom grown for 16 days under control (CTR; 10.5 mM N + 100% W), nitrogen deficit (N; 5.3 mM N + 100% W), water deficit (W; 10.5 mM N + 50% W) or combined nitrogen and water deficit (N + W; 5.3 mM N + 50% FC). The two principal components together (PC1, PC2) explained 81%, in roots, and 87%, in shoots, of the total variance found among treatments. Details on the abbreviations are given in Materials and Methods.

A PCA was performed to establish the relationship among the AOX parameters that were significantly affected by the single or combined N and water deficit treatments. The results yielded two main factors (those with an Eigenvalue > 1), explaining over 81% or 87% of the total variance at root and shoot level, respectively (Figure 7). The first component accounted for 62% and 64% of variance in roots and shoots, respectively, and the second for 19% and 23%, respectively (Figure 7a,b). At the root level, CTR and N-deficit plants behaved similarly, and these showed an opposite behavior to single or combined water deficit plants, mostly because the latter group had a higher activity of CAT and APX enzymes and *CAT2*, *Cu,ZnSOD*, and *APX* expression profiles (Figure 7a). Still, at the root level, the W deficit plants were associated with higher flavonoid concentration and lower antioxidant activity (ABTS), with N + W plants having the opposite behavior regarding these variables (Figure 7a). Interestingly, when analyzing plant responses at the shoot level, plants subjected to combined N + W deficit were different from all the other groups, mainly due to higher anthocyanins concentration, higher activity of CAT, SOD, and APX enzymes, and of *CAT2* and *APX* expression (Figure 7b). However, they shared some similarities with W deficit plants regarding lignin and flavonoid levels, which were higher in these groups, and *CAT1* expression, which was downregulated (Figure 7b).

## 4. Discussion

More often than not, plants do not face single abiotic stresses, but rather a combination of several adverse growth conditions, especially under the current context of climate change and environmental instability. Still, literature describing the physiological and biochemical responses of plants simultaneously exposed to different stresses is still scarce. Works focusing on the effects of combined N + W deficit on tomato plants can provide important and practical data for understanding impactful stress responses in likely environmental scenarios. To the best of our knowledge, this is the first study which focus on a broad analysis of the AOX responses of young tomato plants subjected to single and combined N and W deficits. By studying a set of AOX biochemical endpoints, we showed that tomato plants can modulate their AOX network in order to ensure redox homeostasis, at least in some situations of N and water limitation (Figure 7).

Previous reports have demonstrated that water and/or N deficit severely affect plant performance, leading to a growth reduction in most crops, including tomato (recently reviewed by [5]). In our study, W deficit per se did not induce an evident effect on TDW, whereas in the single or combined N deficit plants, a negative effect was found (Figure 1a), which suggests a higher impact of the N deficit on this trait. Interestingly, dry matter partitioning only responded significantly under water deficit conditions (single or combined), resulting in a decreased shoot–root ratio (Figure 1b), representing a greater investment in the roots to handle the water shortage.

When facing stressful environments, plant often experience a general redox disbalance, capable of downstream affecting other metabolic and physiological processes. It has been reported that ROS can play a dual role in plant metabolism [7]: while at high concentrations, ROS function as a toxic by-product, causing negative lipid membrane damage, at low concentrations, they might be acting as a signal transduction molecule to trigger a stress response, therefore improving non-enzymatic and enzymatic components [6,7,11]. Drought stress has been reported to result in higher MDA content (related to lipid peroxidation) in tomato plants, indicating that under drought stress, the balance of the redox status is disrupted, resulting in ROS accumulation and increased lipid peroxidation of membrane lipids, eventually causing membrane damage [13,39,40,41]. The same was reported for the N deficit [42]. In the present study, although plants under combined N+W deficit showed a significantly lower TDW and shoot–root ratio (Figure 1) compared to CTR and W deficit plants, ROS content and oxidative damage were not significantly affected by any of the applied treatments, as demonstrated by the unchanged values of LP and H_2_O_2_ (Figure 4). Based on this set of results, it seems that mild N and W deficits were perceived by the plant as a signal, helping them to employ defense strategies to better cope with potential future stress episodes. In line with the low levels of H_2_O_2_ found, an increase in the total AOX activity (evaluated by the ABTS Assay) was observed under combined deficit, which might be related with the improvement found in different non-enzymatic factors (such as flavonoids and anthocyanins) (Figure 3) and enzymatic components (SOD, CAT, and APX transcripts and activity) players (Figure 2, Figure 5 and Figure 6).

It is known that non-enzymatic mechanisms play a vital role in counteracting oxidative stress and improving plant stress tolerance [6,7]. Specialized metabolites often display potent AOX activity which allows more efficient scavenging of toxic ROS [43]. In tomato, Sánchez-Rodríguez et al. [44] demonstrated that, after water deficit, cv. “Zarina” increased its AOX activity by upregulating the phenylpropanoids and flavonoids synthesis pathway, together with a lower oxidation index. Our results are in accordance with these findings, as a significantly higher shoot flavonoid content was observed in plants subjected to W deficit (Figure 3d and Figure 7). Previously, it has been suggested that N deficiency also triggers the accumulation of flavonoids via increases in phenylalanine ammonia lyase activity [45]. However, here, no significant differences were found upon exposure to individual N deficit (Figure 3c,d). Interestingly, in shoots, flavonoid accumulation upon exposure to combined N + W deficit reached intermediate levels compared to the single deficits. Thus, it is possible that when facing the combined deficit plants responded by regulating the accumulation of other metabolites. It is known that the production of other secondary metabolites with a photoprotective role, such as anthocyanins and carotenoids, is important in plants’ response to oxidative stress caused by N or water deficit [5]. Accordingly, a higher concentration of anthocyanins was found in plants under N + W (Figure 3f), which may have functioned as ROS scavengers, photoprotectants, and/or stress signals in those plants subjected to the combined deficit [46].

Besides the non-enzymatic mechanisms, the enzymatic component is another crucial part of the defense mechanisms against oxidative stress. SOD, as the first frontline defense against oxidative injury, catalyzes the dismutation of O_2_^−^ and generates H_2_O_2_, which is converted to H_2_O and O_2_ by CAT, with both usually increasing in plants grown under drought stress [47]. In tomato plants, studies have shown that the activity of SOD gradually increases in different tomato cultivars subjected to drought stress [13,39,40,41,48,49,50]. Besides SOD, the activity of CAT and APX have also been shown to increase in tomato plants under prolonged drought stress, from the seedling to the anthesis stage [50,51]. In the present study, APX and CAT activity was only slightly increased in the roots of plants subjected to W deficit, demonstrating a faint response of the enzymatic AOX system associated with mild water deficit. Concerning N deficiency, its effect on the enzymatic AOX system has scarcely been studied in tomato. In general, it has been shown that N deficiency can lead to an overproduction of ROS, particularly of H_2_O_2_ and O_2_^−^, through the excitation of the photosystem II, consequently enhancing the activity of enzymes like POD and SOD [5]. In cucumber leaves of N-deficient plants, the activity of SOD increased by 37% compared to the control, a higher increase compared to CAT and POD [52]. In wheat, plants subjected to N deficiency also exhibited a significant increase in SOD activity (by 205%), with it being the most upregulated enzyme [53]. This set of results is in agreement with the observed increase in SOD activity of our tomato shoots subjected to N deficiency (Figure 5b). Concerning plants subjected to combined N + W deficit, in roots, CAT and APX showed the same pattern of response observed in plants grown under single W deficit, suggesting that at the root level, the W deficit was the main factor contributing to this response (Figure 5a,c,e). Interestingly, at the shoot level, plants grown under co-exposure to both deficits presented the highest activity of SOD, APX and CAT, which contributed to the enhancement of the total AOX activity (Figure 2).

In general, an upregulation of SOD and APX transcripts in roots of plants subjected to N deficiency did not seem to translate into an enhanced enzyme activity, which can suggest the involvement of post-translational modifications of the enzyme’s status. However, it cannot be excluded that, after a longer period of exposure, these increased transcript levels could actually result in higher enzyme activity to ensure cell homeostasis, providing the cell with a large amount of mRNA ready to be translated into proteins. Regarding combined deficit plants, in general, the activity of all the studied enzymes increased both in roots and shoots, along with an upregulation of *Cu,Zn-SOD*, *APX*, and *CAT2* genes in roots and *APX* and *CAT2* genes in shoots.

An integration of all the AOX system-affected traits resulted in a PCA that revealed considerable variability among treatments, especially between tissues (Figure 7). The PCA showed that in roots, N + W deficit plants had a behavior similar to that of plants exposed only to mild W deficit, showing higher activity of CAT and APX enzymes and *CAT2*, *Cu*, *ZnSOD*, and *APX* expression (Figure 7a). In shoots, N + W deficit had a distinct and unique response, having, in general, a higher non-enzymatic and enzymatic activity (Figure 7b). The accumulation of AOX metabolites (e.g., ascorbate and glutathione) alongside the activation of several AOX enzymes (e.g., APX, CAT) was also shown to increase upon the co-exposure of tomato plants to salinity and heat stress) [18] or after the exposure of citrus plants to combined drought and heat stress [54]. The differential behavior between combined N and water deficit and single deficits, as well as between plant organs (shoots and roots) observed in our study, highlights that plants grown under combined stresses generate a specific AOX response that cannot be elucidated from the responses obtained when stressors are applied individually [20,55,56].

Moreover, the mild water deficit level imposed in this study did not impact plant biomass, but it was enough to activate multiple AOX responses in plants grown under combined deficit, possibly making the plants more suited to handling subsequent abiotic stresses. Indeed, a recent study with maize showed beneficial stress memory responses induced by priming the seedlings with combined heat and drought stress, which prompt their defense system to trigger more effective clearance mechanisms against subsequent exposure to these combined stresses [57]. However, the intensity of the N-deficiency needs to be thoroughly adjusted to avoid, as in this study, impairments in plant growth. Flowering and fruit development have been described as highly sensitive stages to water deficits [5,58]. Therefore, complementary studies are necessary to address tomato responses to more severe stress events, as well as to explore other developmental stages.

## 5. Conclusions

This study suggests that tomato plants can employ defense strategies to cope with combined N and water deficit, as demonstrated by the maintenance of the redox homeostasis, with unchanged values of LP and H_2_O_2_ compared with control plants. Accordingly, an enhancement of different non-enzymatic (such as flavonoids and anthocyanins) and enzymatic (SOD, CAT, and APX transcripts and activity) players was observed, contributing to an increased AOX activity, especially in plants under co-exposure to N and water deficit. In conclusion, tomato plants seem to have the ability to modulate their AOX network in order to ensure redox homeostasis in a situation of mild N and water limitation, and the pattern of response is quite distinct among different plant organs and to the one observed in plants exposed to these single deficits. Additionally, our results raise the hypothesis that mild N and water deficits, at least at the beginning of the tomato plant’s vegetative stage, activate relevant AOX responses which in turn might act as a priming strategy for preparing the plants to cope with future abiotic or biotic stresses. However, understanding the full mechanisms involved in the complex interactions between combined stress factors is still in its infancy. Future studies could possibly test the aforementioned hypothesis and test whether the current findings can be extrapolated to more susceptible tomato cultivars, more critical developmental stages as well as to more severe or prolonged stress levels.

## Figures and Tables

**Table 1:** Primer sequences (Forward and Reverse) and annealing temperature (T_ann_.) of target genes used for RT-qPCR analysis.

Gene (Accession Number)	Primer Sequence (5′-3′)		T_ann._
	Forward	Reverse	
*Fe-SOD*	TAA ATA GAG ACT TTG GTT CC	TAT ATT TGC CTC TTA ACC CT	45.6
*Cu,Zn-SOD*	GGC CAA TCT TTG ACC CTT TA	AGT CCA GGA GCA AGT CCA GT	54.7
*APX*	TCT GAA TTG GGA TTT GCT GA	CGT CTA ACG TAG CTG CCA AA	55.4
*CAT 1*	CAA ACA ATG GAC CCC GAG GA	ACT GGG ATC AAC GGC AAG AG	60.3
*CAT 2*	GGG TCT GGT GTC CAC ACA TT	GCA TGG CTG TGA TTT GCT CC	59.0

## Data Availability

Not applicable.
